# Cautery

**Published:** 1882-09

**Authors:** 


### CAUTERY.

A neat, clean and handy way of cauterizing the gum may be done as follows; Dip into a small bottle of nitric acid a small piece of silver wire, shake off any adherent acid and apply the wire at the point of the gum which you desire to cauterize. The wire may be bent before introducing it into the acid, of the shape desired, so as to cauterize just as much of the gum as is desirable —as for instance where a cavity is on the labial surface of an incisor or cuspid and the gum hangs into it and the view is intercepted—by bending the wire in a semi-lunar form and then dipping it in the acid, the gum may be cauterized just to the extent desired, and the operation proceeded with. In cases, too, between the teeth, far back, where it is difficult to use the rubber dam, the gum may be thus cauterized and a flow of blood and saliva stopped sufficiently long for the insertion of an amalgam or other plastic filling.—*Dental Office and Laboratory.*

